# Research and Development Strategy for Future Embryonic Stem Cell-Based Therapy in Japan

**DOI:** 10.31662/jmaj.2018-0029

**Published:** 2020-09-23

**Authors:** Akihiro Umezawa, Yoji Sato, Shinji Kusakawa, Rin Amagase, Hidenori Akutsu, Kazuaki Nakamura, Mureo Kasahara, Yoichi Matsubara, Takashi Igarashi

**Affiliations:** 1 Center for Regenerative Medicine, National Center for Child Health and Development, Tokyo, Japan; 2 Division of Cell-Based Therapeutic Products, National Institute of Health Sciences, Kawasaki, Japan; 3 Organ Transplantation Center, National Center for Child Health and Development, Tokyo, Japan; 4 National Center for Child Health and Development Research Institute, Tokyo, Japan; 5 National Center for Child Health and Development, Tokyo, Japan

**Keywords:** embryonic stem cells, ESCs, regulation, Pharmaceuticals, Medical Devices, and Other Therapeutic Products Act, The Act on the Safety of Regenerative Medicine

## Abstract

Herewith, we review an updated progress of regenerative medical products using human embryonic stem cells (ESCs) in Japan. Two groups from Kyoto University and the National Center for Child Health and Development (NCCHD) established a novel derivation/cultivation system of ESCs for potential application in translational and clinical research. At the first stage of ESC derivation, murine feeder cells have been used in line with Japanese guidelines on public health associated with the implementation of the xenograft. To avoid exposure of ESCs to animal products in culture media, a xeno-free cultivating system has been established. Twelve ESCs (KhES-1, KhES-2, KhES-3, KhES-4, KhES-5, SEES-1, SEES-2, SEES-3, SEES-4, SEES-5, SEES-6, and SEES-7) are now available under a clinically relevant platform for industrially and clinically applicable regenerative medical products. NCCHD submitted an investigative new drug application to the Pharmaceuticals and Medical Devices Agency (PMDA) for using ESC-based products in patients with hyperammonemia due to genetic defects on March 2018 under the Pharmaceutical Affairs Law (now revised to the Pharmaceuticals, Medical Devices, and Other Therapeutic Products Act). Currently, up to ten ESC-based products are being prepared for intractable and rare disorders in Japan.

## Embryonic Stem Cell (ESC) Lines for Regenerative Medical Products in Japan

Human ESCs have high potential to be raw material to produce a variety of cell types due to their pluripotency when compared with somatic stem cells which have limited abilities in differentiation and self-renewal ^(1), (2)^. Once effective and efficient differentiation protocols are established to obtain a target cell, human ESCs are expected to stably supply a major portion of raw cell substrate materials used for manufacturing regenerative medical products. Kyoto University and the National Center for Child Health and Development (NCCHD) strictly complied with the Guidelines for Derivation and Distribution of Human Embryonic Stem Cells ^(3)^ and the Guidelines for Utilization of Human Embryonic Stem Cells ^(4)^ for human dignity to establish ESCs in Japan. In these two guidelines, the basic issues concerning the protection of personal information and protocols of derivation and usage of human ESCs are defined from the viewpoints of bioethics. The guidelines are now for development of novel treatments and pharmaceuticals/medical devices ^(5), (6)^.

Twelve human ESC lines (KhES-1, KhES-2, KhES-3, KhES-4, KhES-5, SEES-1, SEES-2, SEES-3, SEES-4, SEES-5, SEES-6, and SEES-7) are available as a resource of cell substrates to manufacture regenerative medical products at present for clinical usage under the Pharmaceuticals, Medical Devices, and Other Therapeutic Products Act (PMD Act, formerly Pharmaceutical Affairs Law) ^(7), (8), (9)^. KhES-1, KhES-2, and KhES-3 can be maintained in culture for up to 2 years without significant alteration in differentiated capability and karyotypes ^(7)^. Meanwhile, SEES-4, SEES-5, SEES-6, and SEES-7 can also be stably cultivated under xenogeneic-free conditions ^(9)^. SEES-1, SEES-2, and SEES-3 have been established by utilizing murine feeder cells. For a quality evaluation, we thus need to follow “Derivation and Characterization of Cell Substrates Used for the Production of Biotechnological/Biological Products,” “Guidelines on Public Health Infection Issues Accompanying Xenotransplantations,” and “Guidelines on Epithelial Regenerative Therapy Using 3T3J2 Strain or 3T3NIH Strain Cells as Feeder Cells” in order to prevent contamination of feeder cells and transmission of bacteria, fungi, viruses, and prions. KthES11, KthES12, KthES13, and KthES14 cells were established by Kyoto University in a feeder-free culture system to avoid contamination of murine cell components. Suemori has been generating KhESC lines at Kyoto University and has inserted a letter “t” to a name of newly established ESC, which are called KthES-11 cells, because he intends to apply KthES cells for therapeutic purpose in a clinical setting.

## Two Acts on the Regulation of ESC-Based Medical Products

A new regulatory framework to supervise regenerative medicine, i.e., the “Act on the Safety of Regenerative Medicine” and the “Pharmaceuticals, Medical Devices, and Other Therapeutic Products Act,” was enacted in November 2015 ^(10)^. The Act on the Safety of Regenerative Medicine, in place of the Medical Practitioners Act and the Medical Care Act, provides regulations on medical professionals’ practices and clinical studies on regenerative medicine. Under the new “Act on the Safety of Regenerative Medicine,” regenerative medicine is classified into three categories depending on each therapy’s potential risk factor. The risk of regenerative medicine studies using ESCs is considered high; thus, ESC-based therapy will be placed in the high-risk medical technology category (Class I). Regardless of risk factor, all plans of using regenerative medicine must be submitted to the Ministry of Health, Labour and Welfare (MHLW). Moreover, before submitting plans to the MHLW, a certified special committee must submit their opinion on the preliminary plan created by the medical institution that intends to offer ESC-based therapy. This committee fulfills the legal accreditation criterion laid down by the MHLW. Once the committee gives its opinion, regardless of the recommendation, the medical institution may submit their preliminary plan for practicing regenerative medicine along with the committee’s opinion to the MHLW. In addition, medical institutions are obligated to conduct follow-up reports detailing the adverse effects and annual details about their regenerative medicine plan implementation, including the treatment provided, the number of patients treated, and the effectiveness of the treatment to the MHLW and the committee.

## “Donors’ Protection” versus “Patients’ Safety”

We follow two regulations for different legal benefits, namely, “safety and quality” (Guideline on Ensuring the Quality and Safety of Pharmaceuticals and Medical Devices Derived from the Processing of Human Embryonic Stem Cells) and “ethics” (Guidelines on the Derivation of Human Embryonic Stem Cells, Guidelines on the Distribution and Utilization of Human Embryonic Stem Cells) ^(1), (5), (6)^. These guidelines have different standpoints and, therefore, conflict: the former guideline focuses on protection of donors’ personal information and human dignity, and the latter prioritizes safety of raw materials.

## Safety of Raw Materials as Regenerative Medical Products

If human ESC lines are established for the purpose of clinical use, donors need to be selected appropriately. Selection criteria and eligibility of the donors are important. Infection with hepatitis B virus, hepatitis C virus, human immunodeficiency virus, adult human T-lymphotropic virus, or parvovirus B19 shall be ruled out via physician-donor interviews and clinical laboratory tests, such as serological tests and nucleic acid amplification tests. Infection controls at the right time need to be done by retesting, taking into consideration the window period; however, such retesting is not practically allowed for ESCs according to the Guidelines on the Derivation of Human Embryonic Stem Cells ^(5)^. Infection with cytomegalovirus, Epstein-Barr virus, or West Nile virus shall also be ruled out, if necessary, via appropriate clinical laboratory tests. In addition, the eligibility of donors should be assessed whether he or she ever received a blood transfusion or underwent a transplantation procedure. Alternatively, it is conceivable to assess infectious pathogens at intermediate products during differentiation from the viewpoint of ethical and scientific rationality.

## Banking of Intermediate Products during Practical Manufacturing

**Figure 1.**
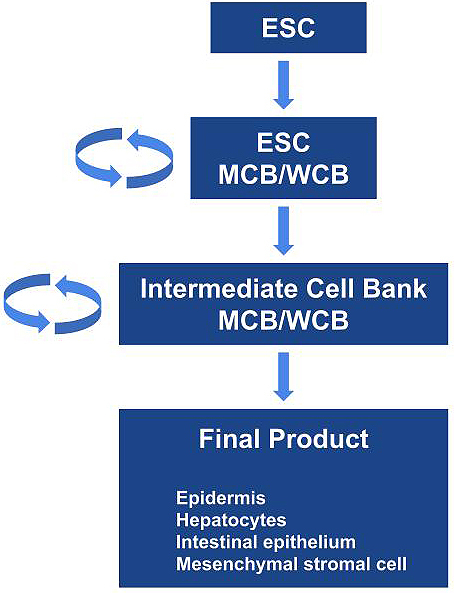
Schematic diagram of intermediate product bank for practical manufacturing

ESCs are considered the most undifferentiated cells similar to inner cells of blastocysts. Thus, the manufacturing process from undifferentiated ESCs to functional differentiated cells or final products is complicated and requires a long timeline, which may be challenging to perform. From the viewpoint of consistency and robustness, the most ideal foundation in the sustainable manufacture of ESC-based products is intermediate cell products/lines that have been well characterized (Figure 1). Preferably, the intermediate products should be stable per se and possess the ability to be propagated under relevant conditions. Furthermore, banks of the intermediate products should be renewed and must be able to differentiate properly into target cells. For certain final products, the proper establishment of sustainable intermediate cell products/lines as a cell bank at the intermediate stage of manufacturing process may be more significant and scientifically rational for the consistent manufacturing of desired safe products in addition to characterization, evaluation, or control of cells at the raw material stage. To minimize issues/concerns as much as possible, the most important concept and measures common to all types of biologics production are to ensure consistency and robustness in the manufacturing process. One of the core technical elements for proper and consistent production of biologics is to establish a base camp, i.e., to prepare production substrates at a relevant stage in the manufacturing process, which is applicable to extensive characterization and control, stable in quality, and from which constant processing to next intermediates and finally to a desired product is achievable.

## ESC-Based Candidate Products in the Development Pipeline

Human ESC-based products have been developed for clinical trials worldwide ^(11), (12), (13), (14), (15), (16), (17), (18), (19), (20)^. Clinical trials using hESC-derived cells for transplantation were identified by exploring the public database (Table 1). We used the search term “human embryonic stem cell” in reviewing “ClinicalTrials.gov,” from the US National Library of Medicine (https://clinicaltrials.gov/) database. We also evaluated original research articles and the sponsor’s websites on the respective clinical trials and/or transplant products for additional information. For example, ESC-derived products are being tested for use to patients with age-related macular degeneration, Stargardt disease, type 1 diabetes, and spinal cord injury in the United States; age-related macular degeneration and Stargardt disease in South Korea, the United Kingdom, China, and Brazil; age-related macular degeneration in Israel; severe heart failure in France; and Parkinson’s disease in Australia. We have filed a new investigational drug application to PMDA for ESC-based regenerative medical products to patients with congenital metabolic disorders and have ESC-derived mesenchymal stromal cells, epidermal cells, intestinal organoids, and hepatocytes in the development pipeline ^(21), (22)^.

**Table 1.** Clinical Trials Using hESC-derived Cells for Transplantation.

Study title	Cell type (product name)	Sponsor/collaborator	Trial location	Disease	Stage of trial	Cell delivery	Status of trial	Registration number	Estimated or actual study completion date	Note/reference
**A. Eye disease**										
Safety and Tolerability of Sub-retinal Transplantation of hESC-Derived RPE (MA09-hRPE) Cells in Patients with Advanced Dry Age-Related Macular Degeneration (Dry AMD)	hESC-derived RPE (MA09-hRPE)	Astellas Institute for Regenerative Medicine	United States	Dry AMD	Phase I/II	Cell suspension	**Completed**	NCT01344993	August 1, 2015	< Progress reports > Lancet. 2015 Feb 7;385(9967):509-16. Lancet. 2012 Feb 25;379(9817):713-20. < Follow-up study > Invest Ophthalmol Vis Sci. 2016 Apr 1;57(5):ORSFc1-9.
Sub-retinal transplantation of hESC-derived RPE (MA09-hRPE) cells in patients with SMD	hESC-derived RPE (MA09-hRPE)	Astellas Institute for Regenerative Medicine	United States	Stargardt’s macular dystrophy (SMD)	Phase I/II	Cell suspension	**Completed**	NCT01345006	August 1, 2015	
Long Term Follow Up of Sub-retinal Transplantation of hESC Derived RPE Cells in Stargardt Macular Dystrophy Patients	hESC-derived RPE (MA09-hRPE)	Astellas Institute for Regenerative Medicine	United States	Stargardt’s macular dystrophy (SMD)	Phase I/II	Cell suspension	Active, not recruiting	NCT02445612	December 1, 2019	Follow-up study for NCT1345006
Long Term Follow Up of Sub-retinal Transplantation of hESC Derived RPE Cells in Patients With AMD	hESC-derived RPE (MA09-hRPE)	Astellas Institute for Regenerative Medicine	United States	Dry AMD	Phase I/II	Cell suspension	Active, not recruiting	NCT02463344	December 1, 2019	Follow-up study for NCT1344993
Safety and tolerability of sub-retinal transplantation of hESC-RPE cells in patients with SMD	hESC-derived RPE (MA09-hRPE)	Astellas Institute for Regenerative Medicine	United Kingdom	Stargardt’s macular dystrophy (SMD)	Phase I/II	Cell suspension	**Completed**	NCT01469832	September 1, 2015	< Progress reports > Ophthalmology. 2018 Nov;125(11):1765-1775.
A Follow up Study to Determine the Safety and Tolerability of Sub-retinal Transplantation of Human Embryonic Stem Cell Derived Retinal Pigmented Epithelial (hESC-RPE) Cells in Patients With Stargardt's Macular Dystrophy (SMD)	hESC-derived RPE (MA09-hRPE)	Astellas Institute for Regenerative Medicine	United Kingdom	Stargardt’s macular dystrophy (SMD)	Phase I/II	Cell suspension	Active, not recruiting	NCT02941991	December 1, 2019	Follow-up study for NCT1469832
A Phase 1b Dose Escalation Evaluation of Safety and Tolerability and a Phase 2 Proof of Concept Investigation of Efficacy and Safety of ASP7317 for Atrophy Secondary to Age-related Macular Degeneration	hESC-derived RPE (ASP7317)	Astellas Institute for Regenerative Medicine	United States	AMD	Phase I/II	Cell suspension	Recruiting	NCT03178149	October 1, 2026	Clinical study of ASP7317, a new cell line to replace MA09-Hrpe
A Phase I/IIa, Open-Label, Single-Center, Prospective Study to Determine the Safety and Tolerability of Sub-retinal Transplantation of Human Embryonic Stem Cell Derived Retinal Pigmented Epithelial (MA09-hRPE) Cells in Patients With Advanced Dry Age-related Macular Degeneration (AMD)	hESC-derived RPE (MA09-hRPE)	CHABiotech (licensed from Astellas Institute)	Korea	Dry AMD	Phase I/II	Cell suspension	Unknown	NCT01674829	April 1, 2016	< Progress report > Stem Cell Reports. 2015 May 12;4(5):860-72. < Progress report of NCT01674829 > JAMA Ophthalmol. 2017 Mar 1;135(3):287-289.
Safety and Tolerability of MA09-hRPE Cells in Patients with Stargardt’s Macular Dystrophy (SMD)	hESC-derived RPE (MA09-hRPE)	CHABiotech (licensed from Astellas Institute)	Korea	Stargardt's macular dystrophy (SMD)	Phase I	Cell suspension	Unknown	NCT01625559	June 1, 2015	
A study of implantation of RPE in subjects with acute wet age-related macular degeneration	hESC-derived RPE (PF-05206388)	Pfizer/University College, London	United Kingdom	Wet AMD	Phase I	Membrane-immobilized monolayer sheet	Active, not recruiting	NCT01691261	December 1, 2019	< Progress report including nonclinical and clinical studies > Nat Biotechnol. 2018 Mar 19. doi: 10.1038/nbt.4114.
Retinal Pigment Epithelium Safety Study For Patients In B4711001	hESC-derived RPE (PF-05206388)	Pfizer	United Kingdom	Wet AMD	Phase I	Membrane-immobilized monolayer sheet	Active, not recruiting	NCT03102138	October 4, 2020	Follow-up study for NCT01691261
Safety and Efficacy Study of OpRegen for Treatment of Advanced Dry-Form Age-Related Macular Degeneration	hESC-derived RPE (OpRegen)	Lineage Cell Therapeutics (former BioTime)/Cell Cure Neurosciences	United States and Israel	Dry AMD	Phase I/II	Cell suspension	Recruiting	NCT02286089	December 1, 2024	https://lineagecell.com/products-pipeline/opregen/ < Nonclinical study > Transl Vis Sci Technol. 2017 Jun; 6(3): 17
Study of subretinal implantation of human ESC-derived RPE cells in advanced dry AMD	hESC-derived RPE (CPCB-RPE1)	Regenerative Patch Technologies	United States	Dry AMD/geographic Atrophy	Phase I/II	Membrane- immobilized monolayer sheet	Active, not recruiting	NCT02590692	June 1, 2023	< Nonclinical study > Graefes Arch Clin Exp Ophthalmol. 2016 Aug;254(8):1553-65. < Progress report of clinical study (phase I/II) > Science Translational Medicine 04 Apr 2018: Vol. 10, Issue 435, eaao4097 http://www.sankeibiz.jp/business/news/180405/prl1804051101040-n1.htm
Clinical Study of Subretinal Transplantation of Human Embryo Stem Cell Derived Retinal Pigment Epitheliums in Treatment of Macular Degeneration Diseases	hESC-derived RPE	Southwest Hospital	China	AMD and Stargardt	Phase I/II	Cell suspension	Active, not recruiting	NCT02749734	December 1, 2019	No available information
Subretinal Transplantation of Retinal Pigment Epitheliums in Treatment of Age-related Macular Degeneration Diseases	hESC-derived RPE	Chinese Academy of Sciences/Beijing Tongren Hospital	China	Dry AMD	Phase I/II	Cell suspension	Recruiting	NCT02755428	December 1, 2019	No available information
Treatment of Dry Age Related Macular Degeneration Disease With Retinal Pigment Epithelium Derived From Human Embryonic Stem Cells	hESC-derived RPE	Chinese Academy of Sciences/The First Affiliated Hospital of Zhengzhou University	China	Dry AMD	Phase I/II	Cell suspension	Recruiting	NCT03046407	December 1, 2020	No available information
Stem Cell Therapy for Outer Retinal Degenerations	hESC-derived RPE	Federal University of São Paulo	Brazil	Dry AMD/wet AMD/Stargardt	Phase I/II	Cell suspension or monolayer in a polymeric substrate	Unknown	NCT02903576	June 1, 2019	No available information
A Safety surveillance study in subjects with macular degenerative disease treated with human ESC-derived retinal pigment epithelial cell therapy	hESC-derived RPE (MA09-hRPE)	Astellas Institute for Regenerative Medicine	United States	Macular degeneration	Phase I/II	Cell suspension	Enrolling by invitation	NCT03167203	December 1, 2029	A long-term (up to 15 years) safety surveillance study
Safety and Efficacy of Subretinal Transplantation of Clinical Human Embryonic Stem Cell Derived Retinal Pigment Epitheliums in Treatment of Retinitis Pigmentosa	hESC-derived RPE	Qi Zhou/Beijing Tongren Hospital	China	Retinitis pigmentosa	Phase I	-	Recruiting	NCT03944239	December 1, 2020	No available information
Interventional Study of Implantation of hESC-derived RPE in Patients With RP Due to Monogenic Mutation	hESC-derived RPE	Centre d'Etude des Cellules Souches	France	Retinitis pigmentosa (due to monogenic mutation)	Phase I/II	-	Recruiting	NCT03963154	December 15, 2021	No available information
**B. Other disease**										
Transplantation of Human Embryonic Stem Cell-derived Progenitors in Severe Heart Failure (ESCORT)	hESC-derived CD15+ Isl-1+ cardiac progenitors	Assistance publique, Hôpitaux de Paris	France	Severe heart failure	Phase I	Cells embedded in fibrin patch	**Completed**	NCT02057900	March 22, 2018	< Nonclinical study > Eur Heart J. 2015 Mar 21;36(12):743-50. < Progress reports of clinical study > Eur Heart J. 2015 Aug 7;36(30):2011-7. J Am Coll Cardiol. 2018 Jan 30;71(4):429-438.
A Safety, Tolerability, and Efficacy Study of VC-01™ Combination Product in Subjects With Type I Diabetes Mellitus	hESC-derived pancreatic precursor cells (VC-01™ combination product)	ViaCyte/California Institute for Regenerative Medicine (CIRM)	United States/Canada	Type 1 diabetes	Phase I/II	PEC-01 cells encapsulated in a medical device	Active, not recruiting	NCT02239354	January 1, 2021	VC-01™, a combination product (PEC-01™ cells + Encaptra® DDS) (see PMID: 29369575) <Manufacturing information > Stem Cells Transl Med. 2015 Aug;4(8):927-31.
One-Year Follow-up Safety Study in Subjects Previously Implanted With VC-01™	hESC-derived pancreatic precursor cells (VC-01™ combination product)	ViaCyte	United States	Type 1 diabetes	Observational study	PEC-01 cells encapsulated in a medical device	Enrolling by invitation	NCT02939118	November 1, 2021	VC-01™ (PEC-Encap™) delivers the PEC-01 pancreatic progenitor cells in a immunoprotective device https://viacyte.com/products/pec%e2%80%90encap-vc-01
A Safety and Tolerability Study of VC-02™ Combination Product in Subjects With Type 1 Diabetes Mellitus	hESC-derived pancreatic precursor cells (VC-02™ combination product, aka PEC-Direct)	ViaCyte	Canada	Type 1 diabetes	Phase I	PEC-01 cells loaded into a delivery device	**Completed**	NCT03162926	February 15, 2018	VC-02™ (PEC-Direct™) delivers the PEC-01 pancreatic progenitor cells in a non-immunoprotective device https://viacyte.com/products/pec-direct
A Safety, Tolerability, and Efficacy Study of VC-02™ Combination Product in Subjects With Type 1 Diabetes Mellitus and Hypoglycemia Unawareness	hESC-derived pancreatic precursor cells (VC-02™ combination product, aka PEC-Direct)	ViaCyte	United States	Type 1 diabetes	Phase I/II	PEC-01 cells loaded into a delivery device	Recruiting	NCT03163511	March 1, 2022	
Safety Study of GRNOPC1 in Spinal Cord Injury	hESC-derived oligodendrocyte progenitors (GRNOPC1/AST-OPC1)	Asterias Biotherapeutics	United States	Spinal cord injury	Phase I	Cell suspension	**Completed** (took over from Geron)	NCT01217008	July 1, 2013	< Nonclinical study > Regen Med. 2015 Nov;10(8):939-58.
Dose Escalation Study of AST-OPC1 in Spinal Cord Injury	hESC-derived oligodendrocyte progenitors (AST-OPC1)	Asterias Biotherapeutics	United States	Spinal cord injury	Phase I/II	Cell suspension	**Completed**	NCT02302157	December 1, 2018	https://www.cirm.ca.gov/our-progress/awards/phase-iiia-dose-escalation-safety-study-ast-opc1-patients-cervical-sensorimotor (You can check progress report on the trial)
A Study to Evaluate the Safety of Neural Stem Cells in Patients With Parkinson's Disease	Human parthenogenetic stem cell-derived neural stem cells(ISC-hpNSC)	Cyto Therapeutics/International Stem Cell Corporation	Australia	Parkinson's disease	Phase I	Cell suspension	Active, not recruiting	NCT02452723	June 1, 2020	< Nonclinical study > Sci Rep. 2016 Sep 30;6:34478.
Safety and Efficacy Study of Human ESC-Derived Neural Precursor Cells in the Treatment of Parkinson’s Disease	Human embryonic stem cells-derived neural precursor cells	Chinese Academy of Sciences/The First Affiliated Hospital of Zhengzhou University	China	Parkinson's Disease	Phase I/II	Cell suspension	Recruiting	NCT03119636	December 1, 2020	< Nonclinical study > Stem Cell Reports. 2018 Jul 10;11(1):171-182.
A Study to Evaluate Transplantation of Astrocytes Derived From Human Embryonic Stem Cells, in Patients With Amyotrophic Lateral Sclerosis (ALS)	Astrocytes derived from human embryonic stem cells (AstroRx)	Kadimastem	Israel	ALS (amyotrophic lateral sclerosis)	Phase I/II	Cell suspension	Recruiting	NCT03482050	August 1, 2020	< Nonclinical study > Stem Cell Res Ther. 2018; 9: 152.
Safety Observation on hESC Derived MSC Like Cell for the Meniscus Injury	hESC-derived MSC-like cell	Tongji Hospital/Chinese Academy of Sciences	China	Meniscus injury	Phase I	-	Active, not recruiting	NCT03839238	September 30, 2020	No available information

AMD: Age-related macular degeneration hESC: Human embryonic stem cell SMD: Stargardt's macular dystrophy RPE: Retinal pigment epithelium DDS: Drug delivery system

## Article Information

### Conflicts of Interest

None

### Disclaimer

Takashi Igarashi is the Deputy Editor of JMA Journal and on the journal's Editorial Staff. He was not involved in the editorial evaluation or decision to accept this article for publication at all.
